# Antibacterial and dermal toxicological profiles of ethyl acetate extract from *Crassocephalum bauchiense *(Hutch.) Milne-Redh (Asteraceae)

**DOI:** 10.1186/1472-6882-11-43

**Published:** 2011-05-26

**Authors:** Raymond S Mouokeu, Rosalie AN Ngono, Paul K Lunga, Martin M Koanga, Alambert T Tiabou, Guy SS Njateng, Jean DD Tamokou, Jules-Roger Kuiate

**Affiliations:** 1 Laboratory of Microbiology and Antimicrobial Substances, Faculty of Sciences, P.O Box 67 Dschang - Cameroon; 2 Laboratory of Biochemistry, Faculty of Sciences, PO Box 24157, Douala - Cameroon; 3 Laboratory of Phytochemistry, Institute of Medical Research and Medicinal Plant Studies (IMPM), P.O Box 6163 Yaounde-Cameroon

## Abstract

**Background:**

The emergence in recent years of numerous resistant strains of pathogenic bacteria to a range of formerly efficient antibiotics constitutes a serious threat to public health. *Crassocephalum bauchiense*, a medicinal herb found in the West Region of Cameroon is used to treat gastrointestinal infections as well as liver disorders. The ethyl acetate extract from the leaves of *C. bauchiense *was evaluated for its antibacterial activity as well as acute and sub-acute toxicities.

**Methods:**

The plant extract was prepared by maceration in ethyl acetate. Its phytochemical screening was done by standard methods. The broth microdilution method was used to evaluate the *in vitro *antibacterial activity. The *in vivo *antibacterial activity of a gel formulation (0.05, 1 and 2% w/v) of this extract was evaluated using a *Staphylococcus aureus*-induced dermatitis in a murine model. Selected haematological and biochemical parameters were used to evaluate the dermal sub-acute toxicity of the extract in rats.

**Results:**

Phytochemical screening of the *C. bauchiense *extract revealed the presence of alkaloids, phenols, tannins and sterols. *In vitro *antibacterial activities were observed against all the tested microorganisms (MIC = 0.04-6.25 mg/ml). Formulated extract-gel (2% w/v) and gentamycin (reference drug) eradicated the microbial infection after five days of treatment. A single dermal dose of this extract up to 32 g/kg body weight (bw) did not produce any visible sign of toxicity. Also, daily dermal application of the *C. bauchiense *extract gel formulation for 28 days did not show any negative effect, instead some biochemical parameters such as alanine aminotransferase (ALT and AST), low density lipoprotein (LDL), high density lipoprotein (HDL) and triglycerides were significantly (p < 0.05) affected positively.

**Conclusion:**

These results indicate that the *C. bauchiense *ethyl acetate extract can be used safely for the treatment of some bacterial infections.

## Background

Plant-based medicaments had served from the onset as the most important therapeutic weapon available for man to fight various human and animal diseases. The exclusive use of herbal remedies to treat and manage ailments continued until the introduction of modern synthetic medicines. The advent of synthetic medicines in the health care system coupled with industrialization, urbanization in most developed countries and the tendency to embrace western culture by the developing countries made the use of herbal products to decline from about the beginning of the 20^th ^century up to the 1970s [1]. However, in recent times, a renewed and growing interest in the use of plant-derived biologically active compounds as drugs or basis in the manufacture of more potent medicaments has been noticed [2]. Plants therefore remain the main natural source of active drugs and are still indispensable in traditional medicine for the treatment of a large number of diseases. Traditional medicines are used by about 60% of the world's population both in the developing countries and developed countries where modern medicines are predominantly used [3].

In Cameroon, like in other developing countries, traditional medicine accounts for more than 80% of rural populace health needs [4]. They are administered in most of the disease conditions over a long period of time without a proper dosage monitoring and consideration of toxic effects that might result from such a prolonged use. Although, a great number of scientific researchers demonstrated activities of several African plants, few of them venture into studying their toxicity.

From field studies, *Crassocephalum bauchiense *appears to be used in folk medicine in the West Region of Cameroon to treat gastrointestinal infections as well as liver disorders. To the best of our knowledge, no scientific evaluation of the therapeutic potentials of this plant has been done. The present work was designed to ascertain the antibacterial activity and safety levels for the dermal administration of ethyl acetate extract of *C. Bauchiense *in the treatment of dermal bacterial infections.

## Methods

### Plant material

*Crassocephalum bauchiense *leaves were collected in Dschang, West Region of Cameroon. The botanical identification was done at the National Herbarium in Yaoundé (Cameroon) by referring to the sample number 7954/SRF/Cam. A voucher specimen of the plant is kept in the Herbarium of the Department of Plant Biology of the University of Dschang under the code number 0033/UDs/PB.

### Microorganisms

The microorganisms used included five ATCC bacterial strains (S*taphylococcus aureus *ATCC 25922, *Enterococcus faecalis *ATCC 10541, *Escherichia coli *ATCC 11775, *Pseudomonas aeruginosa *ATCC 27853, *Salmonella typhi *ATCC 6539) and three clinical isolates (*Escherichia coli, Pseudomonas aeruginosa, Staphylococcus aureus*) obtained from "*Centre Pasteur du Cameroun, Yaoundé*". The strains were maintained at +4°C on agar slants.

### Experimental animals

Experiments were performed using Wistar albino adult rats of both sex 10 to 12 weeks old (200 ± 30 g) and female Swiss albino mice of 6 to 8 weeks old (18 ± 4 g) bred in the animal house of the Department of Biochemistry, University of Dschang, Cameroon. The animals were fed with a standard diet. Food and water were given *ad libitum *to all animals used for the experiments. Animals were maintained at room temperature (22 ± 2°C) and were handled according to standard protocols for the use of laboratory animals. The studies were conducted according to the ethical guidelines of Committee for Control and Supervision of Experiments on Animals (Registration no. 173/CPCSEA, dated 28 January, 2000), Government of India, on the use of animals for scientific research.

### Preparation of plant extract

The leaves of *C. bauchiense *were dried at room temperature for ten days and powdered to coarse particles. Seven hundred grams of the powder were macerated with 5 l of ethyl acetate for two days with frequent stirring. After filtration, the solvent was removed under reduced pressure using a rotary evaporator (45°C) to yield a paste of 71.75 g (10.25%). The phytochemical analysis was performed following standard methods [5].

#### Preparation of the gel formulation of the extract

Shea butter oil was obtained from the seed of Shea nuts collected from Bangwa, West region, Cameroon. Thirty grams of the oil was thawed by heating in a boiling water bath. Bee wax (7.5 g) (bought from a local honey manufacturer) was added and homogenized to yield the vehicle. Sufficient quantities of the ethyl acetate extract of *C. bauchiense *were added to obtain extract-gel concentrations of 0.5, 1.0 and 2.0% w/v.

### *In vitro *antibacterial activity

The *in vitro *antibacterial activity of the extract was performed by determining the minimum inhibitory concentrations using broth microdilution method [6]. Briefly, the stock solution of *C. bauchiense *extract was dissolved in 5% tween 80 in water. Bacterial suspensions of about 1.5×10^8 ^CFU/ml (Mc Farland turbidity standard no. 0.5) were prepared. To obtain the inocula, these suspensions were diluted 100 times in Muller Hinton broth to give 1.5 × 10^6 ^CFU/ml. The antimicrobial susceptibility tests were performed in 96 wells microplates. A serial two-fold dilution of the plant extract was performed to obtain final concentration range of 6.25 to 0.04 mg/ml for the extract and from 128 to 0.0625 μg/ml for the reference drugs in a total volume of 200 μl/well. Each well contained the test substances at a particular concentration and the bacterial suspension (100 μl) in Muller Hinton broth. The plates were incubated at 35°C for 18 h. Growth was monitored colorimetrically using iodotetrazolium chloride (INT). Viable bacteria change the yellow dye of *p*-iodonitrotetrazolium violet to a pink colour. All concentrations at which no visible colour changes were observed were considered as inhibitory concentrations and the lowest of these concentrations was considered as the MIC. The bactericidal concentrations were determined by adding 50 μl aliquots of the preparations (without INT), which did not show any visible colour change after incubation during MIC assays, into 150 μl of extract-free Mueller Hinton broth [6]. These preparations were further incubated at 35°C for 48 hrs and bacterial growth was revealed by the addition of INT as above. All extract concentrations at which no colour changes were observed were considered as bactericidal concentrations. The smallest of these concentrations was considered as the MBC [6].

The assays were carried out in triplicate. Gentamycin and ciprofloxacin were used as positive controls, and 5% tween 80 solution was used as negative control.

### *In vivo *antimicrobial assay

Furs were clipped from about 10% of the body surface area 24 h before the experiment. Infection was established by inoculating the clipped portion, at two different sites, with 0.1 ml of *S. Aureus *suspension (2 × 10^8 ^CFU/ml) prepared from an overnight culture [7]. Infected mice were divided into six groups of five animals each (three control and three test groups). The first control group was not treated, the second and third received vehicle and gentamycin (2%) respectively. The three other groups were treated with gel-extract at 0.5, 1 and 2% (w/v) respectively. Treatment started 48 h after the establishment of the infection by dermal application of 0.05 g of gel-extract once per day for 5 consecutive days. Body weights were measured before inoculation of bacterial suspension and once daily during the experiment period. At the end of the treatment, animals were anaesthetised with chloroform vapour and the skin sample was excised and homogenized in normal saline and further cultured on Mannitol Salt Agar Medium for bacterial count.

### Acute toxicity study

A single dose dermal toxicity was conducted according to the OECD guidelines of toxicity studies [8]. Furs were clipped as described above prior to the application of the test substance. Rats were divided into one control and five treated groups (each group consisted of ten animals including 5 males and 5 females). The control group received vehicle (32 g/kg) while each treated group received the *C. bauchiense *extract gel at 4, 8, 16 and 32 g/kg bw by dermal application. Clinical signs and mortality were observed 15, 30 min and 1, 2, 3 and 6 h after administration of test substances and twice daily for 14 days. Body weights, water and food consumption were recorded throughout the experimental period.

### Sub-acute toxicity study

Furs were clipped from at least 10% of the body surface area of rats 24 h before the experiment [9]. Animals were divided into one control and four treated groups. Each group consisted of ten animals (5 females and 5 males). The control group received vehicle (2400 mg/kg) and each treated group received the *C. bauchiense *extract-gel (30 mg/kg, 300 mg/kg, 1200 mg/kg and 2400 mg/kg) by dermal application for 28 days (once a day).

Observations were made twice each day for motility and mortality. Clinical examinations were made once prior to the first treatment and once weekly. Changes in skin, fur, eyes, mucous membranes and excretions and autonomic activity were noted [9]. At the end of the 21^st ^days of exposure, motor activity, grip strength and sensory reactivity to stimuli of different types were assessed. Animal weight, water and food consumptions were recorded throughout the experimental period.

### Sample collection

On the 28^th ^day of experiment, urine was collected from individual metabolic cages containing animals subjected to overnight fasting [10].

Blood samples were collected by cardiac puncture from ketamine-diazepam anaesthetized rats into heparinised and non heparinised tubes. The non heparinised tubes were allowed to clot and were centrifuged at 3000 rpm for 5 min to obtain the serum. Animals were further sacrificed and used for gross pathological examinations including weight of different organs (liver, kidney, lung, heart and spleen) [11]. Fifteen percent homogenate of liver and spleen tissues from experimental animals were prepared in normal saline solution. The homogenates obtained were centrifuged at 3.000 rpm for 30 min and the supernatants were used for protein quantification [12].

### Biochemical analysis

The serum was assayed for creatinine, aspartate amino transferase (AST), alanine amino tranferase (ALT), total cholesterol, high density lipoprotein (HDL), triglycerides and total protein using commercial kits (Hospitex diagnostic, Roma, Italia). Urine was assayed for total protein and creatinine using the same commercial kits. The liver and spleen homogenates were also assayed for their protein content.

### Haematological analysis

Malassez chamber was used to quantify the total red blood cells (RBCs) and white blood cells (WBCs). Haematocrit was estimated using standard methods [13].

### Histopathological analysis

Immediately after collecting the blood samples, vascular perfusion was performed for hepatic tissue fixation using isotonic saline solution (250 ml) followed by 10% phosphate buffered formalin solution (250 ml). Small pieces of liver were subjected to haematoxylin-eosin staining [14]. Pathological observations were performed on gross and microscopic basis. Histological plates were encrypted for analysis by a histopathologist.

### Statistical analysis

Data were subjected to the one way analysis of variance (ANOVA) and recorded as mean ± SD and where differences exist, means were compared using Waller Duncan test at 0.05 significant level.

## Results

### Antibacterial activity

Phenols, alkaloids, flavonoids, tannins, triterpenes and sterols were identified in the ethyl acetate extract of *C. bauchiense*. This plant extract expressed antibacterial activity on all the tested microorganisms (Table 1). However, this activity was interesting only on *S. aureus *(MIC = 48 μg/ml and MBC = 160 μg/ml). *P. aeruginosa *and *E. coli *(clinical isolate) were the most resistant to the extract (MIC = 3125 μg/ml and MBC = 6250 μg/ml). MICs values were four fold less than the MBC values, indicating that the bactericidal effect of the *C. bauchiense *ethyl acetate extract could be expected.

**Table 1:** Antibacterial activity (MIC, MBC) of the ethyl acetate extract from *C. bauchiense*.

Microorganisms	parameters	extract (μg/ml)	ciprofloxacin (μg/ml)	gentamycin (μg/ml)
*S. aureus *ATCC25922	MIC	48	0.04	4
	MBC	195	0.19	16
*E. faecalis *ATCC 10541	MIC	390	0.09	1
	MBC	1562	1.56	1
*E. coli *ATCC11775	MIC	1562	0.09	16
	MBC	3125	1.56	32
*P. aeruginosa *ATCC27853	MIC	1562	0.04	8
	MBC	3125	0.19	16
*S. typhi *ATCC 6539	MIC	1562	0.19	2
	MBC	3125	0.780	2
*S. aureus*	MIC	781	8	0.25
	MBC	1562	8	0.25
*P. aeruginosa*	MIC	781	1	8
	MBC	3125	16	8
*E. coli*	MIC	3125	8	1
	MBC	6250	8	1

**Figure 1:**
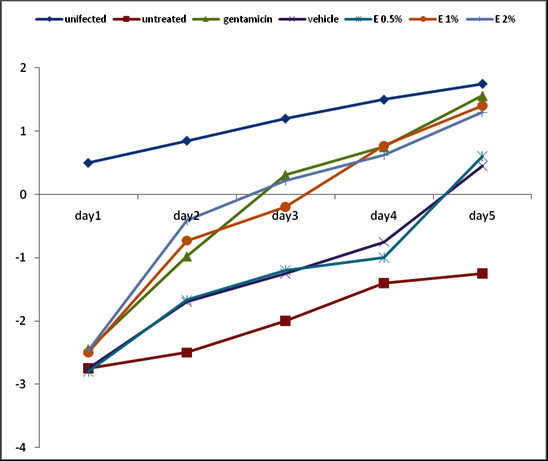
**Effect of the dose of gel-extract on weight gain as a function of time**.

**Figure 2:**
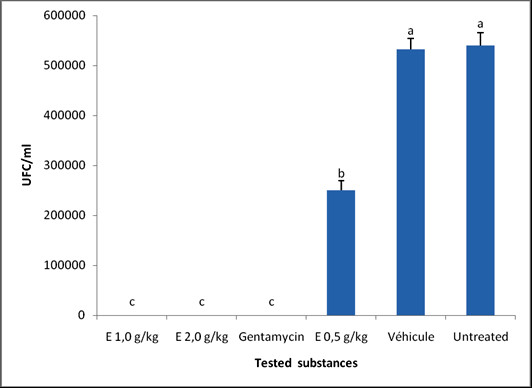
**Effect of gel-extract on bacterial load (UFC/ml) on the 5**^**th **^**day of treatment**.

Animals infected with *S. aureus *showed visible inflammation 48 hours later, characterised by redness and swelling of the skin at the sites of inoculation. Treatment significantly (P < 0.05) reduced the bacterial concentration at the infection sites, with total suppression being noted with the 1 and 2% doses of *C. bauchiense *gel-extract as gentamycin in 5 days (Figure 1). Animals in all groups showed weight loss after infection (Figure 2). From day 1 of treatment, body weight increased gradually with time. On the other hand, the weight gain by the animals of the negative control was lower than that of all the other groups. However, the weight gain by the animals that received the extract at 1 and 2% were comparable (p > 0.05) to gentamycin group, and significantly higher (p < 0.05) than the 0.5% extract-treated group.

### Acute dermal toxicity

No significant variation was noted in rat's body weight at the end of 14 days of experiment between groups. Besides, no changes in skin, fur, eyes, mucous membranes, respiratory and behavioral pattern were observed. In addition, no sign of convulsion, salivation, diarrhea, lethargy, sleep were noted. Up to 32 g/kg bw in both sexes, no death was observed, indicating that the lethal dose fifty (LD_50_) of the *C. bauchiense *extract is higher than 32 g/kg in rats.

### Sub-acute toxicity

#### Effect of extract on relative organ weights

No deaths or signs of toxicity were observed at all doses in the present study. Food and water intake did not show any significant differences between the control and treated groups and between treated groups in both sexes. Relative weights of different vital organs (spleen, kidney, liver, lung and heart) of treated groups were not significantly different from that of control group in both sexes (Table 2).

**Table 2:** Effect of dose and sex on organ relative weights of rats in sub-acute toxicity studies of the ethyl acetate extract from *C. bauchiense*

Sex		Relative organ weight (g/g of tissue)				
	**Dose (mg/kg)**	**liver**	**kidney**	**heart**	**spleen**	**lung**
	
	0	3.34 ± 0.22	0.85 ± 0.02	0.30 ± 0.01	0.39 ± 0.18	0.60 ± 0.09
**Female**	30	3.22 ± 0.37	0.88 ± 0.14	0.29 ± 0.08	0.35 ± 0.12	0.64 ± 0.12
	300	3.18 ± 0.16	0.89 ± 0.07	0.27 ± 0.02	0.38 ± 0.05	0.72 ± 0.21
	1200	3.16 ± 0.12	0.93 ± 0.03	0.28 ± 0.10	0.33 ± 0.04	0.63 ± 0.09
	2400	3.17 ± 0.28	0.94 ± 0.06	0.29 ± 0.03	0.40 ± 0.12	0.73 ± 0.14

	0	3.05 ± 0.22	0.81 ± 0.09	0.29 ± 0.03	0.32 ± 0.06	0.64 ± 0.08
	30	2.85 ± 0.01	0.83 ± 0.07	0.28 ± 0.02	0.37 ± 0.02	0.68 ± 0.02
**Male**	300	3.05 ± 0.34	0.83 ± 0.09	0.26 ± 0.01	0.36 ± 0.02	0.69 ± 0.10
	1200	3.07 ± 0.17	0.93 ± 0.29	0.27 ± 0.02	0.33 ± 0.04	0.69 ± 0.06
	2400	2.96 ± 0.09	0.94 ± 0.03	0.27 ± 0.01	0.39 ± 0.09	0.69 ± 0.06

Data are expressed as Mean ± S.E.M. *n *= 5. Waller-Duncan test (*P *< 0.05).

#### Haematological and biochemical analysis

No significant changes were noted in RBCs, WBCs and hematocrit for both treated male and female rats compared to the control (Table 3). Also, no significant difference in urine and serum creatinine, spleen and hepatic proteins were observed in both sexes. Besides, ALT and AST levels significantly decreased in males and female (Table 4). Lipid indices were affected depending on the animal sex. Indeed, HDL was not affected in female but increased significantly in males from 30 mg/kg bw. In both sexes, the level of LDL and Triglycerides decreased significantly. Total cholesterol was not affected in both sexes. Histopathological analysis showed no deleterious effects on the hepatic tissues of the studied rats of either sex at all the tested doses.

**Table 3:** Haematological parameters of rats in sub-acute toxicity of the ethyl acetate extract from *Crassocephalum bauchiense*

parameter	Control	30 mg/kg	300 mg/kg	1200 mg/kg	2400 mg/kg
**Female**					

RBC (10^6 ^mm^-3^)	9.13 ± 0.30	10.00 ± 0.34	10.46 ± 0.34	10.52 ± 0.32	10. 53 ± 0.37

WBC ^(^10^3 ^mm^-3^)	4.74 ± 0.41	4.80 ± 0.42	4.95 ± 0.32	5.01 ± 0.41	5.30 ± 0.46

PCV (%)	32.25 ± 3.93	32.80 ± 5.30	36.20 ± 4.14	33.40 ± 3.43	33.20 ± 7.39

**Male**					

RBC (10^6 ^mm^-3^)	9.89 ± 0.63	11.10 ± 0.48	11.83 ± 1.01	11.45 ± 1.32	11.92 ± 0.66

WBC ^(^10^3 ^mm^-3^)	5.38 ± 0.56	5.51 ± 0.32	5.6 ± 0.59	6.34 ± 1.34	6.38 ± 0.94

PCV (%)	36.80 ± 3.83	37.00 ± 2.16	37.40 ± 7.16	38.60 ± 7.70	39.00 ± 6.82

Data are expressed as mean ± S.E.M., *n *= 5, RBC = Red blood cell, WBC, = White blood cell PCV = packed cell volume (%).

**Table 4:** Biochemical parameters of rats in sub-acute toxicity of the ethyl acetate extract from *Crassocephalum bauchiense *as a function of treatment dose.

Dosage (mg/kg)	0	30	300	1200	2400
**Female**					

TCHL	79.90 ± 7.56^a^	82.02 ± 7.04^a^	77.10 ± 5.59^a^	82.89 ± 6.64^a^	77.35 ± 5.36^a^
HDL	52.66 ± 3.28^a^	54.95 ± 2.15^a^	52.37 ± 4.34^a^	52.40 ± 5.40^a^	51.75 ± 3.40^a^
LDL	9.00 ± 0.82^a^	10.02 ± 0.71^a^	10.10 ± 1.47^a^	3.75 ± 1.50^b^	0.39 ± 0.05^c^
TG	238.45 ± 7.84^a^	128.69 ± 8.046^b^	125.24 ± 8.22^b^	125.35 ± 7.52^b^	129.31 ± 0.65^b^
UC	79.90 ± 2.33^a^	83.11 ± 2.33^a^	81.51 ± 4.06^a^	84.09 ± 4.24^a^	85.25 ± 4.84^a^
SC	0.84 ± 0.04^a^	0.83 ± 0.02^a^	0.85 ± 0.04^a^	0.85 ± 0.04^a^	0.82 ± 0.02^a^
SP	19.30 ± 2.26^a^	19.99 ± 1.74^a^	21.02 ± 1.61^a^	22.12 ± 1.83^a^	21.26 ± 1.93^a^
UP	2.67 ± 0.52^a^	2.80 ± 0.50^a^	2.69 ± 0.46^a^	2.16 ± 0.37^b^	2.27 ± 0.49^b^
SPL	27.25 ± 1.85^a^	25.85 ± 2.43^a^	27.61 ± 1.78^a^	26.25 ± 1.93^a^	27.48 ± 1.95^a^
HP	33.18 ± 6.68^a^	26.66 ± 4.91^a^	28.88 ± 6.12^a^	30.69 ± 6.57^a^	28.92 ± 3.35^a^
ALT	12.52 ± 0.85^a^	10.20 ± 1.65^b^	7.07 ± 2.28^c^	6.50 ± 2.13^c^	6.68 ± 2.87^c^
AST	55.74 ± 2.61^a^	55.83 ± 2.32^a^	52.57 ± 5.84^a^	40.90 ± 1.02^b^	39.60 ± 1.24^b^

**Male**					

TCHL	116.76 ± 9.97^a^	119.22 ± 13.21^a^	113.04 ± 9.05^a^	117.77 ± 9.04^a^	117.07 ± 10.73^a^
HDL	27.92 ± 2.90^c^	51.28 ± 3.52^b^	51.40 ± 2.09^b^	55.44 ± 1.83^a,b^	57.67 ± 4.88^a^
LDL	8.60 ± 0.54^a^	10.34 ± 0.46^b^	1.96 ± 0.07^c^	1.20 ± 0.44^d^	0.41 ± 0.12^e^
TG	157.96 ± 6.85^a^	118.81 ± 7.81^c^	133.95 ± 7.18^b^	128.31 ± 5.91^b^	125.22 ± 7.38^b^
UC	85.00 ± 2.89^a^	82.68 ± 4.13^a^	82.62 ± 2.14^a^	82.14 ± 3.77^a^	85.53 ± 3.67^a^
SC	0.80 ± 0.05^a^	0.84 ± 0.01^a^	0.80 ± 0.01^a^	0.80 ± 0.05^a^	0.84 ± 0.04^a^
SP	23.33 ± 4.94^a^	23.14 ± 3.98^a^	23.61 ± 5.06^a^	24.04 ± 3.34^a^	23.25 ± 1.92^a^
UP	0.86 ± 0.06^a^	0.82 ± 0.11^a^	0.90 ± 0.15^a^	0.70 ± 0.16^b^	0.70 ± 0.10^b^
SPL	3.62 ± 0.77^a^	3.64 ± 0.73^a^	3.45 ± 0.36^a^	3.60 ± 0.49^a^	3.48 ± 0.29^a^
HP	29.63 ± 1.29^a^	29.87 ± 1.66^a^	27.42 ± 1.67^a^	28.35 ± 2.03^a^	29.72 ± 2.34^a^
ALT	14.81 ± 3.00^a^	10.10 ± 1.43^b^	9.24 ± 1.73^b,c^	7.43 ± 0.58^c,d^	5.03 ± 1.17^d^
AST	54.93 ± 3.04^a^	53.24 ± 5.26^a^	56.16 ± 3.43^a^	38.37 ± 3.39^b^	35.63 ± 2.54^b^

Data are expressed as mean ± S.E.M. *n *= 5. TCHL = *total cholesterol (mg/dl); *HDL = *high density lipoprotein (mg/dl); *LDL = *low density lipoprotein (mg/dl); *TG = *triglycerides(mg/dl); *UC = *urine creatinine(mg/dl); *SC = *serum creatinine(mg/dl); *SP = *serum protein(mg/ml); *UP = *urine protein(μg/ml); *SPL = *spleen protein (mg/g); *HP = *hepatic protein(mg/g); *ALT = *alanine transaminase(U/L); *AST = A*spartate transaminase(U/L).*

Values for a given group in a line followed by same letter as superscript are not significantly different according to Waller Dunkan multiple comparison procedure (at P < 0.05).

## Discussion

The *C. bauchiense *extract was found to express the greatest antimicrobial activities on *S. aureus*, one of the most common causes of skin infections [15]. About 20% of the human population are long-term carriers of *this bacterial specie *[16], responsible for skin infections like impetigo, boils, cellulitis folliculitis, carbuncles, scalded skin syndrome, and abscesses. It can also cause life-threatening diseases such as pneumonia, meningitis, osteomyelitis, endocarditis, toxic shock syndrome (TSS), chest pain, bacteremia, and sepsis [17]. Since the 1 and 2% gel-extracts completely eradicated the *S. aureus*-induced dermatitis in rats within five days as gentamycin; it may be used to treat the above mentioned diseases. The antimicrobial properties of this extract can be linked to the presence of alkaloids, phenols, tannins, triterpenes, flavonoids and sterols. Indeed, members of these phytochemical groups of compounds are known to possess antimicrobial activities [18].

The LD_50 _of the extract was greater than 32 g/kg bw and generally, in both acute and sub-acute toxicity studies, no adverse signs were detected in the treated rats. Based on Hodge and Sterner scale, the *C. bauchiense *extract could be considered as relatively harmless by dermal route [19]. In addition, there were no treatment-related changes in the haematological parameters (hematocrit, RBC and WBC) compared to the control, indicating that *C. bauchiense *extract gel was not toxic to the circulating cells.

Organ-to-body weight ratio, an index often used in toxicological evaluations [3], was not significantly altered by sub-acute treatment. This lends credence to the absence of injuries on the liver, lung, heart, spleen and kidney. A significant decrease of ALT and AST was noted in both sex. Liver damage and its recovery are usually assessed by measuring the level of serum transaminases particularly ALT. Indeed changes in their serum level are biological markers of liver dysfuntioning and/or damage [20]. Thus, *C*. *bauchiense *extract may not be hepatotoxic and may support the current use of this plant species to control liver disorders by local population from the West Region of Cameroon. These results are confirmed by the absence of pathological alteration on liver as reveal by histopathological studies. Similar results were reported with *Crassocephalum vitellinum *[21].

The degree to which elevated blood lipids contribute to heart diseases is determined by their distribution among the various lipoproteins classes. High cholesterol level in the blood is the major cause of cardiovascular disorders [22]. High serum levels of triglycerides and low density lipoproteins are associated with coronary artery disease [23]. We noticed a reduction in the serum levels of triglycerides and low density lipoproteins in animals treated with *C. bauchiense *gel-extract. This suggests that the plant extract may contain hypolipidaemic properties and thus might be interesting in the prevention of cardiovascular diseases development.

The significant increase in the serum level HDL cholesterol further strengthens the fact that the plant extract may be used to reduce the risk factors of cardiovascular diseases. For it is established HDL may exert a protective effect against arteriosclerosis and may promote the mobilization of cholesterol, thereby reducing its deposition in vessel walls [23]. HDL is known to offer some removal mechanism that gets rid of peripheral tissue cholesterol as well as cholesterol from VLDL and LDL.

## Conclusion

The overall results of this study indicate that the *C. bauchiense *ethyl acetate extract can be used safely for the treatment of bacterial infections. Further purification and characterization of the active principles of the extract would provide a better understanding of the antimicrobial mechanism.

## Competing interests

The authors declare that they have no competing interests.

## Authors' contributions

RSM is the field investigator. JRK and RANN designated the study and supervised the work. JRK also revised the manuscript. MMK performed statistical analysis. ATT prepared the plant extract. PKL contributed in field work and in manuscript writing and editing. GSSJ and JDT contributed to the phytochemical studies. All authors read and approved the final manuscript.

## Pre-publication history

The pre-publication history for this paper can be accessed here:

http://www.biomedcentral.com/1472-6882/11/43/prepub
